# Assessing bone mineral changes in response to vitamin D supplementation using natural variability in stable isotopes of Calcium in Urine

**DOI:** 10.1038/s41598-018-34568-4

**Published:** 2018-11-13

**Authors:** Ravi Rangarajan, Surajit Mondal, Prashanth Thankachan, Ramananda Chakrabarti, Anura V. Kurpad

**Affiliations:** 1 Division of Nutrition, St. John’s Research Institute, Bangalore, 560054 India; 2 Centre for Earth Sciences, Indian Institute of Science, Bangalore, 560012 India; 3 Department of Physiology, St. John’s Medical College and Hospital, Bangalore, 560054 India

## Abstract

Osteoporosis is a chronic disease of public health importance, particularly in low and middle income countries. Measuring the bone mineral balance (BMB) in a non-invasive manner, and its response to different interventions, is critical to the definition of optimal strategies for its prevention and management. In this study, we demonstrate the usefulness of natural variability in calcium isotopes (δ^44/40^Ca) of urine and the derived BMB estimates as a biomarker of bone health and its responsiveness to interventions. Vitamin D_3_ is commonly used as a supplement for the prevention and treatment of osteoporosis, along with calcium supplements. We studied the effect of a short term vitamin D_3_ supplementation on changes in urine δ^44/40^Ca and the derived BMB. δ^44/40^Ca before and after the vitamin D_3_ supplementation yielded a statistically significant change (p = 0.050) with a positive δ^44/40^Ca enrichment. The mean derived BMB was net positive (0.04 ± 0.05) in comparison to a net negative value for the control group (−0.03 ± 0.01). These results indicate the potential usefulness of urinary natural δ^44/40^Ca and the derived BMB, which, along with bone mineral density could be used as a sensitive marker for precision in the prevention and treatment of osteoporosis.

## Introduction

Osteoporosis, a chronic condition of public health importance is defined by the World Health Organisation (WHO) as a bone mineral density (BMD) at the hip and/or the spine region that is 2.5 standard deviations below that of healthy adults of the same age^1,2^. It is characterised by a reduction of bone mass and decrease of bone strength, eventually resulting in fragile bones and increased fracture risks^3,4^. The pathophysiology underlying osteoporosis is an imbalance between the process of new bone formation and old bone resorption, resulting in changes in the bone mineral balance (BMB). A net BMB close to zero is expected when there is a homeostatic balance between the process of bone formation and bone resorption^3,5,6^. Changes in the BMB are due to several non-modifiable risk factors like sex, age, ethnicity, genetic makeup and certain modifiable risk factors like sedentary lifestyles, calcium intake and vitamin D deficiencies^7^.

The key nutrients influencing the BMB are Calcium (Ca) and Vitamin D^8,9^. Calcium levels in the body are tightly regulated (2.1–2.6 mmol/L) by the effect of parathyroid hormone (PTH) and calcitonin. Calcitonin promotes the deposition of Ca into bone while PTH does the opposite, in parallel with increasing the formation of Calcitriol (1,25 dihydroxyvitamin D_3_) from Cholecalciferol (Vitamin D_3_) in the kidney^10^ to enhance Ca absorption in the gut. The role of vitamin D in Ca homeostasis is well documented^9,11,12^. Vitamin D is a fat-soluble vitamin and its hydroxylated form 25OHD, best reflects body stores of Vitamin D (i.e. sufficiency or deficiency states)^13^. The classification of 25OHD levels for ascertaining deficiency and sufficiency are slightly different as defined by IOF (International Osteoporosis Foundation) and IOM (Institute Of Medicine). According to IOF (IOM), 25OHD levels of <20 ng/ml (<12 ng/ml) is termed as deficiency, 20–30 ng/ml (12–20 ng/ml) as insufficiency, and levels >30 ng/ml (>20 ng/ml) as sufficiency^14^. We have used IOF reference levels throughout the manuscript to avoid confusion.

Vitamin D deficiency is of immense public health interest as globally it is estimated that more than 50% of the world population has inadequate vitamin D levels^14,15^. In addition, even in tropical countries like India, wherein the exposure to sun is high, the deficiency levels can be as high as 75%^14^. Vitamin D deficiency during puberty and prolonged deficiency along with low dietary Ca intake during the period of peak bone mass development has strongly been associated with later stage osteoporosis and high incidences of fractures in the elderly^7,16^. Rapidly quantifying the efficacy of vitamin D supplementation on total 25OHD levels and BMB, and by extension bone health, would allow for precise titration of the dose and administration of Vitamin D required.

A marker for the early diagnosis of osteoporosis is essential for effective treatment or risk reduction^8^. Currently, this is based on the BMD obtained from Dual Energy X-ray absorptiometry (DXA)^17^. Several biomarkers of bone turnover^6,18^ have also been shown to be useful adjuncts to BMD. However, these indices are not sensitive and accurate enough to reflect changes in bone health at short time periods, for example, at a weekly scale^5,17^ and can provide information on only one of the processes involved in bone metabolism, i.e., either bone resorption or bone formation. In addition, it has also been observed that approximately half of fractures occur in patients with BMD scores that do not meet the diagnostic criteria of osteoporosis^18^. Therefore, there is a need for more relevant markers of bone health, ideally, an adjunct marker to BMD that directly relates to bone metabolism, taking into account the processes of bone resorption and formation. One such potential marker is the BMB derived from natural changes in the stable isotopes of Ca.

Calcium (Ca) has six naturally occurring stable isotopes of which ^40^Ca (96.9%) and ^44^Ca (2.1%) are the two most abundant species, followed by ^42^Ca (0.65%). The variability in the ratio of the two most abundant stable isotopes, expressed as δ^44/40^Ca (Eqn. 1) is defined as,

1 $${{\rm{\delta }}}^{44/40}{\rm{Ca}}=({[{}^{44}{\rm{C}}{\rm{a}}/{}^{40}{\rm{C}}{\rm{a}}]}_{{\rm{sample}}}/{[{}^{44}{\rm{C}}{\rm{a}}/{}^{40}{\rm{C}}{\rm{a}}]}_{{\rm{SRM}}915{\rm{a}}}-1)\times 1000$$

δ^44/40^Ca is reported w.r.t. NIST SRM915a standard in parts per thousand (per mille, ‰) or parts per ten thousand (pptt)^5,19^. Following earlier used conventions, we prefer the use of pptt for the ease of understanding^5^. The use of stable Ca isotopes in metabolic research has been documented earlier^19^, and a recent study established the uniqueness of this marker in a well-planned bed rest study to capture bone resorption rates using an isotope mixing model involving BMB^5,20^. A strong link exists between the stable isotopes of Ca in the body and BMB owing to the process of bone remodeling through the opposing actions of bone forming osteoblasts and bone resorbing osteoclasts. During the process of bone remodeling, there is a preferential intake of lighter isotopes (^40^Ca) into the bone resulting in depletion of heavier isotopes (^44^Ca) in bone tissues and enrichment of heavier Ca isotopes in other body tissues and fluids^5,21^. When the process of bone resorption dominates, characterized by leaching of Ca from bone, the depleted bone signal is reflected within body tissues and in urine^5,20,22^. Urine acts as an ideal reservoir where the depleted/enriched fractions get accumulated, and the natural variability in the stable isotopic ratios of Ca in urine could be used as a non-invasive test to estimate the changes in BMB^5^.

In this study, we demonstrate the usefulness of Ca isotopes in urine and the derived BMB estimates as a direct marker of bone health with potential implications for diseased conditions like osteoporosis. We used vitamin D_3_ supplementation (60,000 IU/week) for a duration of 3 weeks, in male subjects between the age group of 18–35 years and who were vitamin D deficient, as an intervention, replicating the clinical practice of vitamin D and Ca supplementation^23^ for the management of osteoporosis. Changes in Ca isotopes and the derived BMB were then used to evaluate the effect of vitamin D_3_ supplementation.

## Results

### Changes in Serum 25OHD and PTH

**Figure 1:**
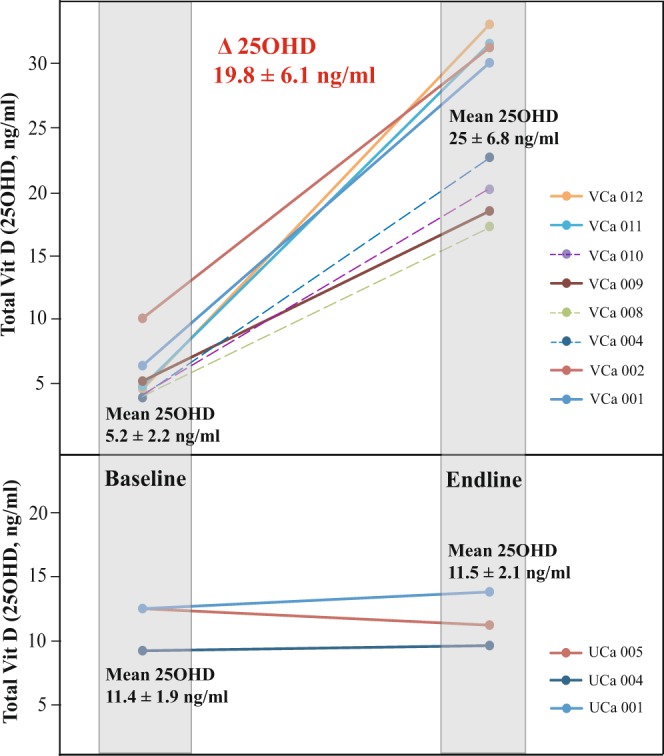
The effect of 60000 IU/week of Vitamin D_3_ (Cholecalciferol) supplementation for a period of 3 weeks on plasma 25OHD levels are displayed. The shaded regions mark the beginning (baseline) and end period (endline) of the supplementation, and the different colored lines are indicative of individual subjects. The control subjects (n = 3, lower panel) to whom intervention was not administered showed no changes in the plasma 25OHD levels, while the intervention group (n = 8, upper panel) exhibited a Δ25OHD of 19.8 ± 6.1 ng/ml. The discontinuous lines in the upper panel indicate the subjects whose baseline 25OHD levels were below our Limits of Detection (LOD >3.9 ng/ml).

The measured baseline 25OHD levels of all the subjects, prior to intervention, ranged from 3.9 ng/ml to 12.5 ng/ml (n = 11). Out of these, 3 subjects had plasma 25OHD levels below our achievable Limits of Detection (LOD; <3.9 ng/ml) and LOD values were fixed as baseline values for calculation and inference purposes. The mean baseline and endline plasma 25OHD concentrations in the intervention group (n = 8) was 5.3 ± 2.2 ng/ml and 25 ± 6.8 ng/ml, with a mean change in 25OHD level (Δ25OHD) of 19.8 ± 6.1 ng/ml (n = 8) and with all the subjects in the intervention arm showing an increase in plasma 25OHD levels (Fig. 1). In the control group, the mean baseline and endline vitamin D levels were 11.4 ± 1.9 ng/ml and 11.5 ± 2.1 ng/ml respectively, whilst remaining largely unchanged. In parallel with 25OHD change, the plasma PTH concentration changed from 64.2 ± 23.9 at baseline to 43.1 ± 13.7 pg/ml at endline in the intervention group. In the control group (n = 3), the corresponding baseline and endline PTH concentrations were 57 ± 28.4 and 73 ± 33.3 pg/ml respectively (Fig. S1). The mean Δ25OHD of 19.8 ± 6.1 ng/ml resulted in a mean PTH change (Δ PTH) of −17.7 ± 11.7 pg/ml (Table S1).

### Changes in Ca isotopic composition and its relation to Dietary Ca intake

**Figure 2:**
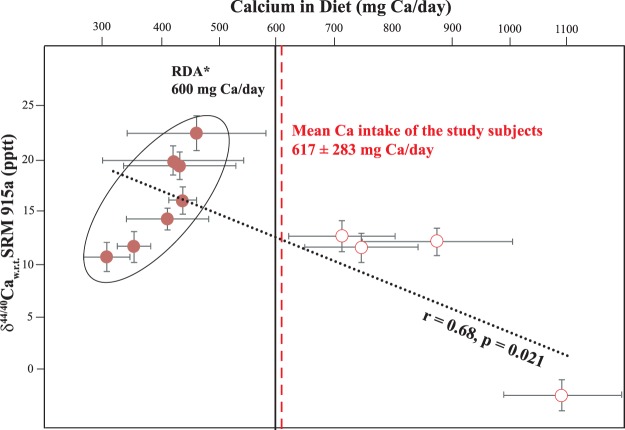
Baseline variability of δ^44/40^Ca in urine of all the subjects (n = 11) are plotted against Ca intake obtained from dietary recalls. A weak but significant correlation indicating depletion in δ^44/40^Ca with increased intake of Ca in diet is seen, with presence of two distinct dietary clusters. The mean Ca intake along with the *Recommended Dietary Allowance^24^ for the study group is also given for reference. Within the low Ca intake cluster (marked in the figure), δ^44/40^Ca and the amount of Ca exhibit a positive correlation.

The baseline δ^44/40^Ca of the subjects, prior to allocating them to different groups, ranged from −2.4 to 22.4 pptt with a mean value of 13.5 ± 6.5 pptt (n = 11, Table S1). At baseline, only one of the eleven subjects exhibited a negative δ^44/40^Ca value (−2.4 pptt) and the mean δ^44/40^Ca of the study group with the exclusion of this subject was 15.1 ± 4.1 pptt. The spread in the overall δ^44/40^Ca was quiet large (1σ = 6.5 pptt) and in order to understand this large spread, we compared it with the dietary Ca intake as a dependence of human Ca isotopic ratios on total Ca intake and dietary Ca composition have been proposed^22,24^. The mean dietary Ca intake for the study group was 617 ± 283 mg Ca/day, just above the recommended daily allowance (RDA) of 600 mg Ca/day for Indian population^25^. The relationship between δ^44/40^Ca and dietary intake of Ca within our study group is shown in Fig. 2. δ^44/40^Ca exhibited a significant correlation (r = 0.68, p = 0.021, n = 11, Fig. 2) with the dietary Ca intake and the subject who exhibited the most depleted δ^44/40^Ca had the highest amount of dietary Ca intake (δ^44/40^Ca = −2.1 pptt, Ca intake of 1092 mg Ca/day). This is expected as higher intake leads to more available Ca for bone remodeling process and hence more of depleted Ca fractions passes into the urine^19,22,24^.

We observed a significant relationship between δ^44/40^Ca and dietary intake, but the dependence was not as strong as expected and previously understood^22,24^. Detailed analyses of the dietary Ca intake data showed that two distinct clusters exist within our study group. There was a high dietary Ca intake cluster with a mean of 857 ± 172 mg Ca/day (n = 4) and a low dietary Ca intake cluster with a mean of 404 ± 54 mg Ca/day (n = 7). The low dietary Ca intake cluster was falling well below the RDA^24^ (Fig. 2, Table S1) and within this cluster, an inverse relationship seems to exist between δ^44/40^Ca and dietary Ca intake. This inverse relationship seems to be controlled by subjects with least Ca intake within this cluster and more detailed studies are essential to understand this inverse relationship in the low intake cluster. Furthermore, the correlation between dietary Ca intake and natural Ca isotopic variability should be interpreted with extreme caution, as there exists enormous difference in sensitivity of the two methods.

**Figure 3:**
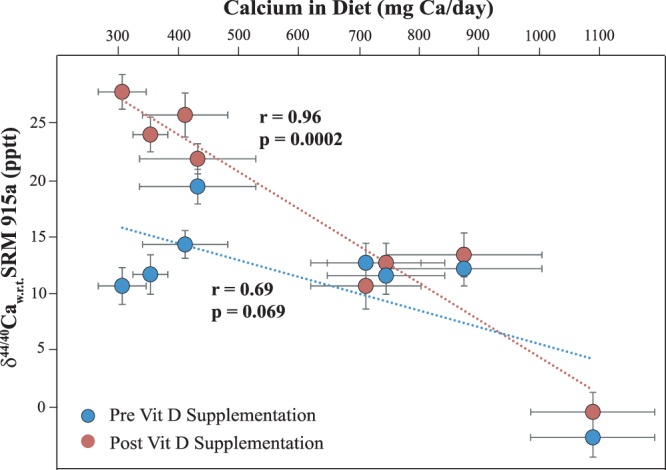
δ^44/40^Ca values pre and post Vitamin D_3_ supplementation is plotted against dietary Ca intake of the subjects who participated in the intervention. It is important to note that upon Vitamin D_3_ supplementation, a stronger dependency between δ^44/40^Ca in urine and dietary Ca is seen. The implication of this observation from the context of Ca bio-availability and its implications for osteoporosis management is discussed in detail in the text.

The mean Δ25OHD of 19.8 ± 6.1 ng/ml and Δ PTH of −17.7 ± 11.7 pg/ml in the intervention group, resulted in a significant change in the δ^44/40^Ca (Δ δ^44/40^Ca) of the subjects (Table S1). A paired t-test of the δ^44/40^Ca values pre and post vitamin D_3_ supplementation in the intervention arm yielded a significant p-value (p = 0.050, n = 8) with a correlation coefficient of r = 0.70. In the intervention arm, the mean baseline δ^44/40^Ca was 11.3 ± 6.1 pptt and mean endline δ^44/40^Ca was 16.9 ± 9.4 pptt (n = 8). The higher spread of δ^44/40^Ca values seen in post supplementation mean could be due to variable absorption rates, mediated by variable increase in vitamin D levels across subjects. Within the control group, similar changes in δ^44/40^Ca were not observed (Table S1). In addition, in the intervention arm prior to supplementation, there existed a weak relationship between δ^44/40^Ca and dietary Ca intake (r = 0.69, p = 0.069, n = 8, Fig. 3). Upon supplementation, the same relationship showed stronger dependence with higher significance (r = 0.96, p = 0.0002, n = 8, Fig. 3). This drastic improvement in the correlation between δ^44/40^Ca and dietary Ca intake upon supplementation would be due to better absorption and bio-availability of dietary Ca owing to increase in 25OHD levels. The changes in the measured δ^44/40^Ca seen here upon intervention is clearly due to increase in bio-available Ca, and this should eventually result in a positive change in the subjects BMB measure.

### Vitamin D3 supplementation and BMB changes

Our study population was restricted to active healthy adult male individuals within the age group of 18 to 35 years and hence we expected a net positive BMB values centered on zero with very less spread. On the contrary, the mean BMB of the control subjects was −0.03 ± 0.007, indicating a net low negative BMB (Table 1). The major reason for the observed negative BMB in the control group would be the low bio-available Ca through diet, as their net dietary Ca consumption (441 ± 20 mg Ca/day) was well below the RDA, in combination with their low 25OHD levels (11.4 ± 1.9 ng/ml). All the subjects in the intervention group, except for one, showed a net positive BMB upon Vitamin D_3_ supplementation. The BMB of the subjects who took up the intervention was 0.04 ± 0.05, with values ranging from −0.02 to 0.11 (Table S1). The obtained BMB values of the intervention group was significantly different from the BMB values of the control group (p = 0.002, Table 1). Within the supplementation group, the low Ca intake cluster showed higher enrichment in Ca isotopes  (Δ^44/40^Ca) resulting in a significant change in the BMB in comparison to the control group (p = 0.009, Δ^44/40^Ca = 10 ± 6 pptt, BMB = 0.078 ± 0.037, Table 1). The high Ca intake cluster showed a moderate, but significant (p = 0.026, Table 1) net BMB of 0.011 ± 0.022.

**Table 1:** The baseline and endline values of 25OHD, along with Dietary Ca, the calculated enrichment in Ca isotopes (Δ^44/40^Ca) and the derived BMB are displayed.

	Diet Ca (mg Ca/day)	Baseline 25OHD (ng/ml)	Endline 25OHD (ng/ml)	Δ^44/40^Ca (pptt)	BMB^a,b^
Control (n = 3)	441 ± 20	11.4 ± 1.9	11.5 ± 2.1	−2.7 ± 0.5	−0.031 ± 0.007
Low Ca Intake (n = 4)^+^	376 ± 57	5.5 ± 3.1	22.9 ± 6.0	10 ± 6.0	0.078 ± 0.037^†^
High Ca Intake (n = 4)^+^	857 ± 171	5.4 ± 0.9	24.3 ± 8.2	0.1 ± 1.8	0.011 ± 0.022^*^

The control subjects without intervention exhibited a net negative BMB and net negative Δ^44/40^Ca, while the intervention group showed a net positive BMB and positive Δ^44/40^Ca.

^+^3 week supplementation with 60000 IU/week of Cholecalciferol (Calcirol, Cadilla Pharma. Ltd.).

^a^p = 0.002 (All Intervention subjects Vs Controls).

^b^BMB values for low and high Ca intake significantly different from zero.

^†^p = 0.009, (Low Ca Intake Vs Controls).

*p = 0.026 (High Ca Intake Vs Controls).

Among the subjects who took the supplementation, only one subject didn’t show any positive shift in the mean BMB and the BMB value of this subject was very similar to that of control subjects. Closer examination into the dietary recalls revealed that this subject had high levels of consumption of hot beverages like tea or coffee (5–6 cups a day) with a daily Ca consumption of ~700 mg Ca/day. It is well known and documented that presence of tannins and polyphenols in beverages inhibit the absorption of most 2+ ions such as Fe, Zn, Ca etc. Thus, we believe, although the supplementation of Vitamin D_3_ increased the 25OHD levels (from 4.8 to 31.6 ng/ml), increased absorption of Ca did not occur in this subject due to the inhibitory effects of increased consumption of tannins and polyphenols^26,27^.

## Discussion

The 3 week intervention lead to vitamin D sufficiency only in 50% of the individuals and the remaining 50% were still in deficient or insufficient state. All the subjects whose baseline 25OHD levels were below our LOD (discontinuous lines; Fig. 1) fell into this category, indicating that their baseline values could be much less than the assumed LOD value. The possible reasons for this lack of attaining sufficiency status could be the short 3 week intervention period we followed in place of the 8 week recommended period^28^ or could be due to poor Ca dietary constitution of the subjects. We also observed that prior to the intervention, the mean PTH levels of the subjects in the intervention arm were towards the higher side of the normal range (64.2 ± 23.9 pg/ml; Fig. S1; normal range is 10 to 65 pg/ml^29^) and upon intervention there was a decrease in the mean and scatter of PTH levels (43.1 ± 13.7 pg/ml; Fig. S1). This is clearly indicative of the effectiveness of the given intervention and this was reflected as significant changes in BMB and in turn bone metabolism.

The urinary Ca isotopic composition in humans is largely unknown and very few studies have documented its variability^5,19^. δ^44/40^Ca values of urine are expected to be more enriched (positive) in healthy individuals, in line with the process of selective uptake of ^40^Ca by bone tissues and our baseline δ^44/40^Ca data showed a similar pattern. In our study group, the intervention with vitamin D_3_ resulted in significant changes in the endline δ^44/40^Ca values. Prior to supplementation, within the intervention arm, there was a weak relationship (p = 0.069) between δ^44/40^Ca and dietary Ca intake, and the same relationship became significant (p = 0.0002) upon supplementation. This significant change in the δ^44/40^Ca and dietary Ca intake was mainly due to the response shown by the low Ca intake cluster within the intervention arm (Fig. 3). It is important to note that the dietary Ca intake is predominantly through the consumption of milk and its products, and in the low Ca intake cluster, the dietary diversity of Ca sources may have been quite poor. In addition, the relationship between δ^44/40^Ca and dietary Ca strongly depends upon the Ca absorption rates in the intestines and very few studies have been carried out till date to understand variability in Ca absorption rates and in turn the Ca bioavailability across populations. More studies in this direction along with studies involving groups with homogenous diet types, will help in better understanding this relationship as Ca supplementation is an inherent part of osteoporosis management strategies.

The implications of low dietary Ca intake on bone mass and its mineral balance has not yet been thoroughly understood in Asian population and the general understanding is that higher 25OHD levels cannot compensate for low dietary Ca intake^16^. In our study, Δ^44/40^Ca was highest in subjects with the least 25OHD levels at baseline (>3.9 ng/ml) and least Ca intake in the entire study group (356 ± 53 mg Ca/day, n = 3). The measured Δ^44/40^Ca of this group was 13.4 pptt and we believe this high enrichment was seen due to better mobilization of Ca with increased 25OHD levels. These subjects (Δ^44/40^Ca = 13.4 ± 3 pptt, baseline 25OHD >3.9 ng/ml, n = 3) collectively showed a net positive BMB of 0.10 ± 0.01, the highest among the subjects in the intervention arm. In addition, the intervention dose we gave was high (60000 IU/week), and the subjects with the least Vitamin D status at baseline (below detection limit) showed maximal changes upon intervention, very much in line with earlier studies^30,31^. Furthermore, this indicates that in subjects with low vitamin D levels and low dietary Ca intake, a condition common in osteoporotic patients, δ^44/40^Ca of urine and the derived BMB can be used as a novel bio-marker for understanding progressive changes in bone health upon treatment with vitamin D/Ca supplementation. In addition, it directly points at the possibility of using the natural variability in Ca isotopes to study and develop dose responses to vitamin D supplementation, and its interaction with an individual’s Ca status.

Comparing the BMB of the control and intervention groups, the observed change in BMB from net negative (−0.03 ± 0.01) to net positive (0.04 ± 0.05) values shows the effect of Vitamin D_3_ supplementation on bone metabolism and health. The natural variability in the net BMB of a population is unknown and the variability in our control group was the only means to establish the intra-individual variability of BMB. In the control group, a net negative BMB (−0.03 ± 0.01) was observed and prolonged sustenance of this negative BMB will result only in a decrease in the subjects BMD measure and bone health. To understand if BMB is a sensible measure of BMD, we looked into the available BMD data of the subjects in the intervention arm. The post-intervention BMB was most positive (0.11) for the subject with highest Z-score (3σ) and lowest BMD value (0.652). In addition, the same subject had the least intake of dietary Ca (307 ± 40 mg Ca/day) along with lowest vitamin D levels (<3.9 ng/ml, below LOD) at baseline. This observation strengthens the relationship between BMB and BMD that has been documented earlier^19^ and further indicates the potential usefulness of BMB obtained from δ^44/40^Ca as a potent marker that could be used along with BMD for prognosis of osteoporotic treatments. More detailed longitudinal study involving the relationship between δ^44/40^Ca, BMB and BMD may prove to be very effective and useful from the perspective of osteoporosis management. Detailed study on a larger scale is also required to know the temporal and population level variability in BMB and such a study would help in establishing natural Ca isotope variability in urine as a non-invasive tool for estimating osteoporotic risks at population level and for identifying high risk populations.

## Conclusion

In here, we showed the usefulness of the natural variability in Calcium isotopes of urine and the derived BMB estimates for understanding the effect of vitamin D_3_ supplementation on bone health with potential implications for osteoporosis management. The baseline δ^44/40^Ca correlated well with the dietary Ca intake data and this relationship seems to be positively correlated for high Ca intake groups and negatively correlated for low Ca intake groups. The mean increase in 25OHD levels resulted in a mean decrease in the PTH levels along with enrichment in Ca isotopes and positive changes in net BMB. Comparing the BMB of the control and intervention groups, the observed change in BMB from net negative to net positive values showed the effectiveness of Vitamin D supplementation, and it’s direct and immediate effect on bone health. In addition, maximum changes in δ^44/40^Ca and maximum positive BMB upon vitamin D_3_ supplementation was observed in subjects with the least 25OHD levels to start with, a condition common in osteoporotic patients. These results indicate that the natural variability in δ^44/40^Ca of urine and the derived BMB from it can be used as a novel bio-marker for bone health and could be used for estimation of osteoporotic risks. Understanding the prognosis of clinical treatments in osteoporotic conditions and its effective management should also be possible with the developed method, provided the method could be quantitatively validated with classical methods like DXA.

## Methods and Materials

### Study Design and Statistical Analyses

In this study, we characterized the natural variability in Ca isotopes in healthy subjects with vitamin D deficiency and the changes in BMB upon Vitamin D intervention in the same group of subjects. In total, 21 healthy subjects were screened at St. John’s Medical College and Hospital, and ~80% of the subjects met our inclusion criteria (Vitamin D deficiency; below 20 ng/ml or 50 nmol/l)^28^. Baseline urine samples were collected from all the subjects and subsequently they were divided into an intervention arm and a control arm containing twelve and four subjects respectively. Only four subjects were allotted to the control group as the primary purpose of the control group was only to know the natural BMB variability. In the intervention arm, a dose of 60000 IU/week of Vitamin D_3_ (pre-vitamin D_3_, activated 7-dehydro cholesterol, Calcirol, Cadilla Pharmaceuticals Ltd.) was recommended for a period of 3 weeks and the subjects were followed up through the duration of intervention. Of the twelve in the intervention arm, four dropped out before the completion of the intervention and in the control arm, one subject dropped out. The data for all the subjects who completed the study are only presented and discussed in here. The study was approved by the Institutional Ethics Committee at St. John’s Medical College and Hospital, India (Ref #169/2017) and informed consent was obtained from all the participants. All the reported methods were performed in accordance with the relevant guidelines and regulations approved by the committee. Statistical comparisons on the obtained data to infer correlations and significances, along with t-tests were performed on Microsoft Excel.

### Dietary Recall

Dietary recall was done for all the subjects to get an estimate on the amount of Ca in diet. The recommended daily intake of Ca for healthy adult males as recommended by Indian Council of Medical Research is 600 mg/day^25^. The multi-pass 24 hr dietary recall carried out, looked at consumption patterns for 3 days during the intervention period. The 3 days consisted of 2 inconsecutive week days and 1 weekend following the standard methodology of 24 hour dietary recall. Standard measuring cups and spoons were used to estimate consumption of portion sizes of each item^32,33^.

### Sample Collection

Urine and blood samples were collected from the subjects at baseline and endline of the study. Fasting urine samples were collected in sterile containers (50 ml) and aliquots were made into 20 ml sterile falcon tubes in the laboratory after collection. The tubes were sealed with parafilm to prevent contamination with ambient dust and stored in −80 °C. In parallel to urine sample collection, 5 ml of fasting blood samples were collected in EDTA tubes. The collected blood samples were immediately centrifuged and the plasma samples stored for biochemical assays.

### Biochemical Assays

Bio-chemical determination of 25OHD and Para-Thyroid Hormone (PTH) levels were done on all the subjects at baseline and endline. The analyses were done on a Roche e411 instrument (Roche Diagnostics, Indianapolis, US) using an Electro-chemiluminescence binding assay method. The Limits of Detection (LOD) for PTH and 25OHD using this method was 1.20 pg/ml (0.13 pmol/l) and 3.9 ng/ml (9.7 nmol/l) respectively. The inter-day coefficient of variation in level 1 and level 2 standards (PreciControl Varia, Roche Diagnostics) used for 25OHD determinations were 1.28 and 1.65% respectively, while the intra-day coefficient of variation in level 1 and level 2 standards were 3.33 and 4.12% respectively.

### Sample Processing and TIMS analyses

10 ml of the collected urine samples, containing ~1 mg of Ca, were acid digested in a microwave digestion system (MARS system, CEM Corp.) using double distilled HNO_3_ (Emsure brand, Merck) and 30% H_2_O_2_ (Emsure brand, Merck). Upon initial digestion, the samples were dried to obtain a pellet devoid of any organics. Complete digestion was then achieved by treatment with aqua-regia (1:3 volume of HNO_3_ and HCl, Merck) in pre-acid washed Teflon reaction vessels. Subsequently, column purification were done in a class 100 clean room at Centre for Earth Sciences, Indian Institute of Science, Bangalore. ^43^Ca-^48^Ca double spike was added to the sample containing approximately 10 μg of Ca, and the Ca fractions were separated using ion-exchange chromatography involving Bio Rad AG50W-X8 (200–400 mesh) resin and 2.5 M HCl based acid eluent scheme. Isotopic measurements were carried out using Thermo Fisher Triton Plus multi collector Thermal Ionization Mass Spectrometry (MC-TIMS) following established analytical procedure^34^. All Ca isotopic data (δ^44/40^Ca) are presented with respect to NIST SRM 915a and our analytical uncertainty (2 SD) was better than ±1pptt (or ±0.1‰), as determined by multiple measurements of NIST SRM915a, SRM915b and seawater NASS 6 standard^34^.

### Ca Isotope Model for deriving BMB

In our calculation of BMB we used an updated Ca isotope mass balance model^5^. The model uses the changes in urinary Ca isotopes to calculate BMB, taking into account renal and hepatic fractionation. The input parameters for the described model are the fractionation factor of Ca isotopes during bone mineralization (ε^44/40^Ca_bone_), the renal Ca isotope fractionation factor (ε^44/40^Ca_urine_), the rate of intestinal absorption of dietary Ca (F_diet_), the mass of skeletal Ca (M_bone_) and the background rate of skeletal remodelling (F_bone_). We used values of 15.8 for and 20.2 pptt for ε^44/40^Ca_bone_ and ε^44/40^Ca_urine_ respectively based on the ε^42/40^Ca values prescribed in the model^5^ and using a mass dependency factor of 2.05^35^. Assuming an average skeletal mineral mass of 3,000 g and a Ca content in bone mineral of 32.2%, we estimated M_bone_ to be 966 mg^5^. The average measured dietary Ca intake obtained based on 3 day dietary recall (2 weekdays and 1 weekend day) was 617 ± 283 mg Ca/d. Accounting for the bioavailability of Ca in the typical diet of 27%^36^, we estimated that ~165 mg Ca/d was absorbed in the intestines and this value was used in the model as F_diet_.

In the existing mass balance model, a value of 500 mg Ca/day was fixed as F_bone_^5^, based on remodelling rate obtained from healthy American volunteers and this estimate may not be applicable for the Indian population studied here. Indians have long been known to consume less amounts of dietary Ca^16^ and the average measured dietary Ca intake for our subjects during the study period was 617 ± 283 mg Ca/day. In contrast, in the control population used for developing the BMB model^5^, the average consumption was 1450 ± 170 mg Ca/day. Studies on African and Asian population with mean dietary Ca consumption below ~400 Ca/day, have shown that increased Ca intake results in increased bone mineral status, possibly in association with decreased bone remodelling space^16,37^. In addition, data on skeletal remodelling using Ca isotopes are unavailable for Indian population and we believe a lower bone remodelling rate in line with low dietary Ca consumption is more realistic. Hence, we assumed a remodelling rate of 250 mg Ca/day for the BMB calculations in the current study and a variable remodelling rate of 250 ± 100 mg Ca/day did not significantly alter the observed trends in BMB.

## Electronic supplementary material

Supplementary Figure S1 and Table S1
