# Supplementary material for: Assessing bone mineral changes in response to vitamin D supplementation using natural variability in stable isotopes of Calcium in Urine
Source: Sci Rep. 2018 Nov 13;8:16751. doi: 10.1038/s41598-018-34568-4 (PMC6233152; doi:10.1038/s41598-018-34568-4)
Supplement: Supplementary file 1 — Supplementary Figure S1 and Table S1 [file 41598_2018_34568_MOESM1_ESM.doc]

**Supplementary Information**

**Assessing bone mineral changes in response to vitamin D supplementation using natural variability in stable isotopes of Calcium in Urine**

Ravi Rangarajan^1*^, Surajit Mondal^2^, Prashanth Thankachan^1^, Ramananda Chakrabarti^2^, Anura V. Kurpad^1,3^

*^1^Division of Nutrition, St. John’s Research Institute, Bangalore – 560054, India*

*^2^Centre for Earth Sciences, Indian Institute of Science, Bangalore – 560012, India*

*^3^Department of Physiology, St. John’s Medical College and Hospital, Bangalore – 560054, India*

** Correspondence:* [*ravi.r@sjri.res.in*](mailto:ravi.r@sjri.res.in)

**Figure S1:** Effect of 3 week supplementation of 60000 IU/week of Vitamin D_3_ (Cholecalciferol) on plasma PTH levels is displayed. The shaded regions mark the beginning (baseline) and end period (endline) of the supplementation period, and different colored lines indicative of individual subjects are provided. The control subjects (n=3, lower panel) to whom intervention was not administered showed a mean increase in PTH levels, while the intervention group (n=8, upper panel) exhibited a mean decrease in PTH levels with ΔPTH of -17.7 ± 11.7 pg/ml. The discontinuous lines in the upper panel indicate the subjects whose baseline 25OHD levels were below our Limits of Detection (LOD >3.9 ng/ml). One of the key points to note is that along with the mean, the spread in the PTH levels also decreased in the intervention arm, while in the control arm it increased.


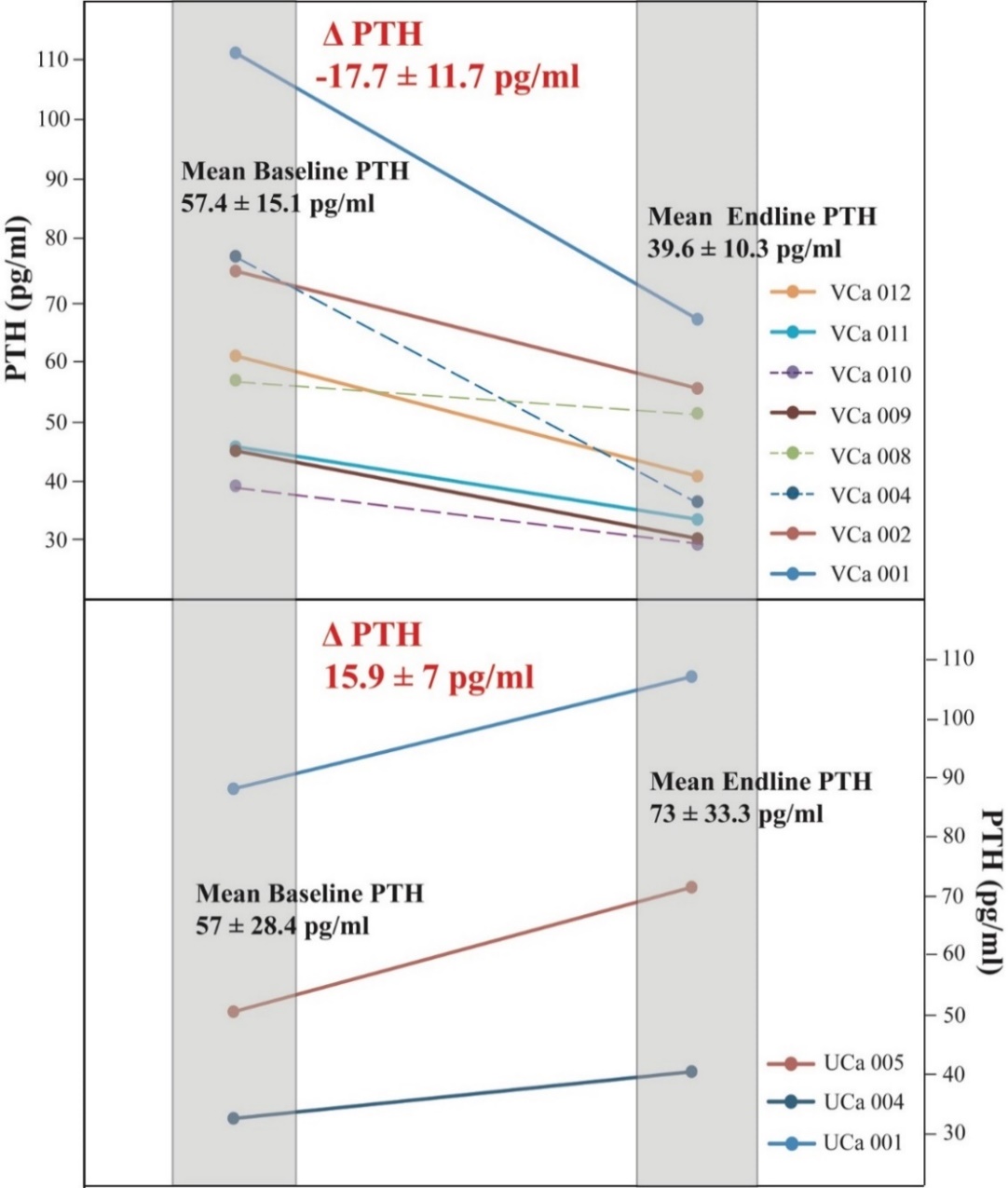


**Table S1:** The baseline and endline values of 25OHD, PTH and δ^44/40^Ca along with dietary Ca intake are shown. The calculated enrichment in Ca isotopes (Δ^44/40^Ca) and the BMB derived using this enrichment are also displayed alongside. The control subjects without intervention exhibited a net negative BMB and net negative Δ^44/40^Ca, while the intervention group showed a net positive BMB and positive Δ^44/40^Ca.

| **Sub ID** | **Diet Ca (mg Ca/day)** | **Baseline** | | | **Endline** | | |  | |
| --- | --- | --- | --- | --- | --- | --- | --- | --- | --- |
|  |  | **25OHD (ng/ml)** | **PTH (pg/ml)** | **δ^44/40^Ca (pptt)** | **25OHD (ng/ml)** | **PTH (pg/ml)** | **δ^44/40^Ca (pptt)** | **Δ^44/40^Ca**  **(pptt)** | **BMB** |
| VCa - 001 | 877 ± 130 | 6.4 | 112.2 | 12.2 ± 1.5 | 30.1 | 67.3 | 13.4 ± 1.9 | 1.2 | 0.016 |
| VCa - 002 | 433 ± 97 | 10.1 | 75.4 | 19.3 ± 1.5 | 31.3 | 55.6 | 21.7 ± 1.3 | 2.4 | 0.024 |
| VCa - 004 | 412 ± 71 | <3.9 | 77.9 | 14.3 ± 1.2 | 22.7 | 36.5 | 25.5 ± 1.9 | 11.2 | 0.089 |
| VCa - 008 | 354 ± 29 | <3.9 | 57.0 | 11.7 ± 1.7 | 17.3 | 51.4 | 23.8 ± 1.5 | 12.1 | 0.090 |
| VCa - 009 | 747 ± 98 | 5.2 | 45.1 | 11.6 ± 1.6 | 18.5 | 30.3 | 12.7 ± 1.7 | 1.1 | 0.014 |
| VCa - 010 | 307 ± 40 | <3.9 | 39.2 | 10.7 ± 1.6 | 20.2 | 29.3 | 27.5 ± 1.5 | 16.8 | 0.110 |
| VCa - 011 | 713 ± 92 | 4.8 | 45.8 | 12.7 ± 1.7 | 31.6 | 33.5 | 10.7 ± 2 | -2.0 | -0.020 |
| VCa - 012 | 1092 ± 108 | 4.6 | 61.1 | -2.4 ± 1.7 | 33.1 | 40.8 | -0.2 ± 1.7 | 2.2 | 0.032 |
| **Mean** | **617** | **5.3** | **64.2** | **11.3** | **25.6** | **43.1** | **16.9** | **5.6** | **0.044*** |
| **SD** | **283** | **2.1** | **23.9** | **6.1** | **6.6** | **13.7** | **9.4** | **6.7** | **0.046** |
| UCa - 001 | 462 ± 120 | 12.5 | 88.1 | 22.4 **±** 1.9 | 13.8 | 107.0 | 19.3 ± 1.5 | -3.1 | -0.037 |
| UCa - 004 | 423 ± 122 | 9.2 | 32.5 | 19.7 ± 1.6 | 9.6 | 40.4 | 16.9 ± 1.7 | -2.8 | -0.032 |
| UCa - 005 | 438 ± 24 | 12.5 | 50.5 | 16.0 ± 1.5 | 11.2 | 71.5 | 13.9 ± 1.7 | -2.1 | -0.024 |
| **Mean** | **441** | **11.4** | **57.0** | **19.4** | **11.5** | **73.0** | **16.7** | **-2.7** | **-0.031** |
| **SD** | **20** | **1.9** | **28.4** | **3.2** | **2.1** | **33.3** | **2.7** | **0.5** | **0.007** |
